# From Lab to Shelf: Gelatin-Based pH Sensors Revolutionizing Food Packaging

**DOI:** 10.3390/gels11050327

**Published:** 2025-04-27

**Authors:** Ruirui Wang

**Affiliations:** 1 College of Chemistry and Chemical Engineering, Qinghai Normal University, 38 Wusi West Road Xining, Xining 810008, China; wangruirui34@qhnu.edu.cn; Tel.: +86-1399-7280-624; 2 Key Laboratory of Advanced Technology and Application of Environmental Functional Materials in Qinghai, Xining 810008, China

**Keywords:** gelatin, pH sensors, smart packaging, food monitoring

## Abstract

The development of multifunctional smart food packaging has garnered considerable attention in research. Gelatin exhibits outstanding characteristics, featuring remarkable gel strength, molecular binding affinity, excellent colloidal dispersibility, low solution viscosity, sustained dispersion stability, and significant water retention properties. Gelatin-based film is ideally suited for the developing simple, portable, and rapid pH sensors, owing to its satisfactory biocompatibility, biodegradability, biosafety, affordability, and facilitation of easy handling and usage. This paper aims to explore the challenges and opportunities relating to gelatin-based pH sensors. It begins by outlining the sources, classifications, and functional properties of gelatin, followed by an analysis of the current research landscape and future trends related to intelligent indicators and active carriers. Subsequently, potential research directions for gelatin-based pH sensors are proposed. Using a literature analysis, it can be concluded that novel gelatin-based smart packaging represents the future of food packaging. It is hoped that the paper can provide some basic information for the development and application of gelatin-based smart packaging.

## 1. Introduction

The food supply chain, spanning from production to consumption, faces numerous challenges, encompassing microbial contamination, chemical residues, and issues related to both personal and environmental hygiene [1]. Food safety represents a critical global public health concern [2,3], with multiple critical points in the production process posing potential risks of contamination or unsafe practices. Ensuring proper storage, physicochemical and microbial stability, and appropriate cooking methods are essential control measures to safeguard food safety [4]. Inadequate assessments of food quality pose significant risks to human health and contributing to food waste. To address these challenges, robust monitoring systems are required to detect contaminants such as pesticides, antioxidants, heavy metals, and bacterial pathogens, among others [5]. Food packaging play a crucial role in reducing food waste, as it is estimated that approximately 130 million tons of food are lost annually worldwide due to inadequate packaging [5]. As improved living standards continue to reshape consumer demands, the functionality of packaging has expanded significantly beyond their traditional role of protection [6,7,8]. Among the emerging trends, the development of multifunctional smart food packaging have garnered considerable attention in research [9,10,11,12,13], reflecting the growing need for innovative solutions to enhance food safety and sustainability.

Smart packaging are designed to provide a range of advanced functionalities, including detection, monitoring, tracking, and communication, while simultaneously offering real-time information and alerts regarding potential threats to food quality [14]. Despite these technological advancements, smart packaging currently represents only 5% of the global packaging market, indicating significant untapped potential for further development and commercialization [15]. Among the diverse functionalities being explored, real-time monitoring of food quality changes has garnered considerable research interest [16,17]. It is widely recognized that food spoilage often involves the production of specific acidic or alkaline compounds, leading to alterations in the internal pH of the packaging environment. As a result, the development of pH sensors has emerged as a promising non-destructive approach for detecting and tracking changes in the internal conditions of packaged food. By exhibiting visible color changes, these materials provide consumers with essential information related to food storage and preservation, such as ripeness, gas or volatile compound content, temperature, and overall food quality [18,19]. The primary objective of these innovations is to enhance food safety, improve product quality, and extend shelf life, thereby addressing critical challenges in the food supply chain.

Global statistics indicate that slaughterhouses produce approximately 10 million tons of biomass waste annually, presenting significant challenges for resource management and environmental sustainability [20]. The extraction and reuse of gelatin from this biomass waste have emerged as a viable strategy to address both resource inefficiency and environmental pollution [21]. Gelatin, a hydrolyzed derivative of collagen sourced from animal skin, bones, and connective tissues, contains all essential amino acids except tryptophan [22]. Compared to natural collagen, gelatin is widely regarded as one of the most versatile biomaterials due to its polar structure, readily available tripeptides, and high solubility [23]. Its cost-effectiveness, abundance, eco-friendliness, and biocompatibility have led to its extensive use in various industries, including food [24,25,26], cosmetics [27,28,29], pharmaceuticals [30,31,32,33,34,35], and biomedicine [36,37,38,39]. In addition to these applications, gelatin films exhibit exceptional gas and oil barrier properties, as well as biodegradability. Unlike plant protein-based films, which often exhibit coloration, gelatin films are colorless and transparent, making them particularly suitable for pH sensors. Their transparency ensures that they do not interfere with the color changes in pH indicators, a critical feature for effective monitoring [40]. The development of gelatin-based pH-sensors are expected to align with and meet the increasingly stringent regulatory requirements of global markets.

## 2. Classification, Sources and Functional Properties of Gelatin

### 2.1. Sources of Gelatin

Gelatin is primarily sourced from biomass waste generated by mammals, poultry, and fish. Currently, approximately 46% of the gelatin available on the global market is derived from pig skin and cartilage [41], followed by bovine hides and bones, as illustrated in Table 1. Compared to bovine gelatin, pig gelatin contains higher concentrations of glycine (Gly), proline (Pro), and arginine (Arg). Mammalian gelatin is particularly preferred for its superior gelling, such as gel strength and viscosity, as well as its excellent film-forming capabilities, which surpass those of gelatin from other sources [42]. However, the use of mammalian gelatin is subject to certain religious and cultural restrictions, limiting its applicability in specific markets [43].

In recent years, the extraction of gelatin from fish and other marine species has gained considerable attention [44,45,46,47,48]. Fish gelatin exhibits functional properties comparable to those of pig gelatin, positioning it as a promising alternative. However, fish gelatin contains lower levels of amino acids, such as proline (Pro) and hydroxyproline (Hyp), which results in reduced thermal stability compared to mammalian gelatin [49]. Additionally, fish gelatin demonstrates inferior gelling properties and rheological performance relative to mammalian gelatin [50]. Similarly, poultry gelatin closely resembles mammalian gelatin in terms of amino acid composition, secondary structure, and molecular weight [51], making it a viable and sustainable alternative for various industrial applications.

The global gelatin market is projected to grow significantly, with an estimated value of USD 5 billion by 2025 and USD 6.7 billion by 2027, reflecting an annual growth rate of 9.29% [52]. This growth underscores the increasing demand for gelatin across diverse industries and highlights the importance of developing sustainable and versatile sources to meet market needs.

### 2.2. Classification of Gelatin

Gelatin is a water-soluble polypeptide obtained through the hydrolysis of collagen via acid, alkali, or enzymatic processes [53]. Its structure is characterized by repeating amino acid sequences such as Gly-Pro-Y, Gly-X-Hyp, and Gly-Pro-Hyp [54]. The structural integrity of gelatin is primarily maintained by intra- and inter-molecular hydrogen bonding, along with electrostatic interactions. During gelatin production, raw materials undergo treatment with dilute acids or alkalis, which breaks down the native collagen into single α-chains, covalently bonded β-chains (comprising two α-chains), and γ-chains (comprising three α-chains). The extraction process parameters, including pH, temperature, and extraction time, significantly influence the molecular structure and weight of gelatin, thereby determining its functional properties and applications [55]. Based on the extraction method employed, gelatin can be classified into two types: type A (produced via acid hydrolysis) and type B (produced via alkali hydrolysis). The transformation of collagen into gelatin involves significant alterations in the amino acid composition, resulting in marked differences between gelatin and native collagen. During the extraction of type B gelatin, alkali treatment promotes the deamination of glutamine into glutamic acid (Glu) and asparagine into aspartic acid (Asp). Consequently, type B gelatin contains notably higher concentrations of Glu and Asp than type A gelatin [41]. The isoelectric point (pI) of gelatin also varies depending on the extraction method, with type A gelatin exhibiting a pI between 8 and 9, while type B gelatin has a pI ranging from 4.8 to 5.4 [54].

### 2.3. Properties of Gelatin

Gelatin is primarily formed from 18 intricate amino acids, among which Gly, Pro, and Hyp collectively account for nearly 57% of its composition. Its molecular structure comprises both single and double helical configurations, which exhibit pronounced hydrophilic characteristics. These structural features contribute to its remarkable gel-forming capacity and strong binding affinity [56]. Gelatin exhibits desired biocompatibility due to the presence of the cell-adhesive Arg-Gly-Asp (RGD) sequence within its molecular structure [57]. The stability of gelatin’s triple-helical conformation is predominantly maintained by Hyp, where the hydrogen atom of Gly forms intramolecular hydrogen bonds with the carboxyl oxygen, reinforcing the helical framework [43,58]. Additionally, water molecules facilitate further stabilization by forming hydrogen bonds with Hyp, enhancing the mechanical strength and structural integrity of films. The gelatin network is further stabilized by abundant functional groups (–NH_2_, –COOH, –OH) [59,60], which contribute to its crosslinked structure, endowing the gelatin-based materials with appropriate stability against gases and moisture. Moreover, approximately 11% of amino acid are consists of hydrophobic residues (Ala, Met, Val, Leu, Ile), whose interactions significantly influence the gel point and gel strength [43]. As a polyampholyte, gelatin contains 13% cationic (Arg, Lys) and 12% anionic (Asp, Glu) amino acids, enabling strong electrostatic interactions. Environmental factors such as temperature, ionic strength, and pH influence conformational flexibility of gelatin, allowing for rapid structural transitions between ordered helices and disordered coils. This adaptability results in the dynamic self-adjustment of gelatin’s morphology and physicochemical properties, granting the gelatin-based materials exceptional flexibility, reversibility, and conformability to diverse surfaces.

Gelatin exhibits outstanding characteristics, featuring remarkable gel strength, molecular binding affinity, excellent colloidal dispersibility, low solution viscosity, sustained dispersion stability, and significant water retention properties [56]. Gelatin possess high potential for commercial application as smart food packaging through their associated and unique characteristics.

### 2.4. Biodegradation of Gelatin-Based Film

Gelatin is traditionally derived from by-products of livestock with high reproductive rates, mainly from cattle (cattle source) and pigs (pig source). Compared to petroleum-based synthetic alternatives, its production offers notable environmental advantages, including a reduced carbon footprint due to lower CO_2_ emissions [61]. At the same time, gelatin can be degraded into amino acids by enzymes after fulfilling its function without generating environmentally harmful byproducts. Increasing environmental awareness in recent decades has stimulated the advancement of new gelatin-based biodegradable films derived from renewable resources. As an eco-friendly alternative, gelatin-based films significantly reduce the environmental footprint associated with conventional non-biodegradable plastic waste [62]. Consequently, gelatin-based film is ideally suited for the development of simple, portable, and rapid pH sensors, owing to its satisfactory biocompatibility, biodegradability, biosafety, affordability, and facilitation of easy handling and usage [63].

## 3. Development of Gelatin-Based pH Sensors

**Figure 1:**
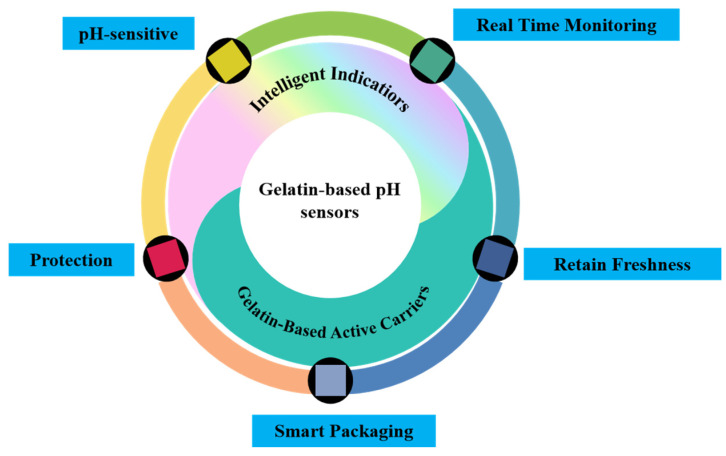
The composition of gelatin-based pH sensors.

Gelatin-based pH sensors are composed of two primary components, an intelligent indicator and an active carrier [52], as illustrated in Figure 1. These sensors are capable of monitoring environmental changes within the packaging in real-time, thereby providing visual information regarding food quality. Using visible indicators, they enable consumers to track and rapidly assess food quality, offering a reliable and non-invasive method for ensuring the freshness and safety of packaged foods.

### 3.1. Active Carriers

The active carrier in pH sensors must provide a sensitive, safe, and stable environment for the intelligent indicator. Gelatin, a low-cost and widely available Generally Recognized as Safe (GRAS) food additive, serves as an ideal carrier for various bioactive compounds, including polysaccharides, nanoparticles, and polyphenols, owing to its excellent oxygen barrier and film-forming. Biodegradable gelatin carriers not only protect packaged food from oxidation, thereby extending shelf life, but also help maintain an appropriate relative humidity within the packaging environment [64]. However, pure gelatin carriers have several limitations, including weak mechanical properties, high hydrophilicity, poor thermal stability, and limited applicability [65]. Studies indicate that the tensile strength of mammalian gelatin-based films ranges from 2.40 to 63.25 MPa [66,67,68]. When in contact with high-moisture foods, pure gelatin films exhibit poor water resistance due to their high moisture absorption. Additionally, gelatin lacks inherent biological activity, making pure gelatin films susceptible to degradation by microorganisms, UV radiation, and oxidative substances, which can compromise food quality. As a result, the use of unmodified gelatin films in practical applications is not recommended.

Cross-linking modification which encompassing physical crosslinking, chemical crosslinking, and enzymatic crosslinking has proven to be the most effective approach for enhancing the properties of gelatin-based films. Reactive modifications of the active groups (-COOH, -NH_2_, -OH) on the gelatin molecules can impart the desired stability to gelatin-based films. In the context of food storage and transport, an ideal modification method should allow for user control over both preservation and degradation times according to specific needs. Nevertheless, most currently employed crosslinking methods for gelatin are irreversible [69].

**Figure 2:**
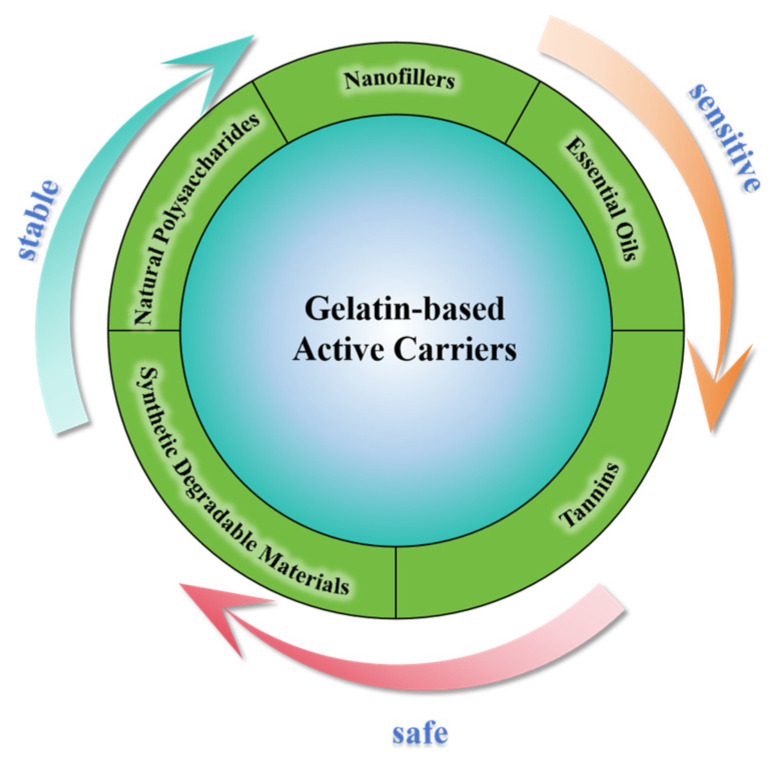
Functional fillers into gelatin matrices.

It is crucial that gelatin-based pH sensors not only ensure the safety of packaged food, but also provide adequate stability to the gelatin carrier. Consequently, the modification for gelatin-based carriers must be safe, efficient, non-toxic, and environmentally friendly. In recent years, the incorporation of functional fillers into gelatin matrices to develop multifunctional active films has emerged as a significant research focus [15,64]. As is shown in Figure 2, functional fillers are critical for the preparing multifunctional gelatin-based pH sensors, as the synergy between functional fillers and gelatin can enhance the composite materials with desired properties. Natural biopolymers, including polysaccharides, nanofillers, essential oils, and tannins, along with biodegradable synthetic polymers, can be integrated into the gelatin matrix to produce smart packaging [70]. The incorporation of active components, including antimicrobial agents, antioxidants, and UV shielding agents, improves the functionality of the films, thereby extending food shelf life, enhancing sensory attributes, and increasing the safety of packaged foods [71,72,73,74].

#### 3.1.1. Natural Polysaccharides

Natural polysaccharides, such as cellulose, starch, chitosan, sodium alginate, carrageenan, and agar, are abundant, renewable, and cost-effective, making them widely utilized in the food industry [75,76,77,78,79]. These polysaccharides can be categorized as cationic polysaccharides or anionic polysaccharides. The incorporation of natural polysaccharides into gelatin for composite modification addresses the issue of poor mechanical properties in gelatin films, thereby enhancing film-forming, biocompatibility, and controllable biodegradability [80,81]. Gelatin-based packaging that integrates natural polysaccharides can prevent the loss of nutrients, flavor, and moisture from packaged food, thus improving the safety of packaged food.

Chitosan is a natural cationic polysaccharide. For example, a radical grafting method was employed to incorporate gallic acid into chitosan. The modified chitosan was subsequently combined with gelatin using hydrogen bonding and electrostatic interactions, resulting in the formation of a stable polyelectrolyte complex and producing a gelatin/chitosan pH-sensitive film [82]. The findings indicate a significant improvement in the water solubility of gallic acid-modified chitosan. This polyelectrolyte complex enhances the encapsulation efficiency of active substances and the stability of natural dyes, making the newly developed dual-functional gelatin-based composite film suitable for preserving food and monitoring food quality. Among natural polysaccharides, starch is particularly well-suited for the preparation of smart packaging. Starch consists of amylose and amylopectin. The ratio of amylopectin significantly influences the mechanical properties of the composite films [79]. Currently, research on gelatin for liquid food smart packaging is limited due to its strong hydrophilicity. A portable sensor capable of accurately measuring milk quality parameters was developed [83], including freshness and potential adulteration. This sensor utilizes a gelatin/starch composite film as a carrier for color-changing polymers, producing films sensitive to hydrogen peroxide, hexavalent chromium, salicylic acid, and pH. These color-changing films were integrated with QR code technology to create a milk testing card that visually reflects milk quality based on color changes. The results demonstrate that the sensor can simultaneously detect three different preservatives (H_2_O_2_, salicylic acid, and Cr(VI)) while monitoring milk freshness. Each sample point on the smart film exhibits color changes directly related to the concentration of the target analyte, enabling users to obtain detailed monitoring data by scanning the QR code. This sensor is low-cost, environmentally friendly, and responsive, offering promising applications in the field of milk quality control.

#### 3.1.2. Nanofillers

Nanofillers provide several advantages, including high specific surface area, flexibility, biocompatibility, ease of modification, and antimicrobial properties. They are widely employed to enhance the mechanical properties, thermal performance, and water vapor barrier capabilities of composite films [84,85,86,87]. The incorporation of nanoparticles into gelatin matrices not only addresses challenges such as poor mechanical performance, brittleness, and processing difficulties associated with gelatin films, but also ensures the safety and quality of food during storage and transportation.

The addition of inorganic nanoparticles, such as titanium dioxide (TiO_2_) [88], silicon dioxide (SiO_2_) [89,90], copper sulfide (CuS) [91], zinc oxide (ZnO) [92], and carbon dots (CDs) [62,93] can significantly enhance the mechanical properties of gelatin films while also endowing them with excellent antibacterial properties, water vapor barrier capabilities, and thermal stability. For instance, Zinc oxide nanoparticles and mulberry extract were incorporated into a gelatin/chitosan matrix through hydrogen bonding to develop a novel pH-sensitive film for real-time monitoring of pork freshness [94]. The inclusion of zinc oxide nanoparticles markedly improved the water vapor barrier of the gelatin-based film. Black rice bran anthocyanins were incorporated into a matrix composed of gelatin and chitin nanocrystals to create a pH-sensitive film with antioxidant activity [95]. The chitin nanocrystals conferred excellent antibacterial properties and thermal stability to the gelatin film, enabling it to visually indicate the freshness of high-protein foods such as shrimp and ribbonfish through color changes. Notably, the film with a lower anthocyanin content demonstrated increased sensitivity to basic volatile compounds released during storage. In another study, copper-based metal–organic frameworks (Cu-MOFs) were integrated into a gelatin/polyvinyl alcohol blend film [96]. MOFs are unique porous nanomaterials formed by coordinating metal ions with organic ligand clusters. The addition of Cu-MOFs enhanced the tensile strength, water resistance, and UV shielding properties of the gelatin film, while also imparting antioxidant and antibacterial capabilities. During freshness monitoring, MOFs nanofillers with high surface areas can adsorb volatile organic amines, thereby enhancing the colorimetric response. The interaction between nanoparticles and gelatin bestows gelatin-based films with excellent antibacterial properties, indicating significant potential for the development of smart packaging [97]. However, the incorporation of nanoparticles considerably increases the cost of gelatin-based films, presenting a challenge that must be addressed.

#### 3.1.3. Essential Oils

Essential oils are natural plant extracts characterized by volatility, containing various active compounds with antibacterial, antioxidant, anti-allergic, and anti-inflammatory properties. However, these oils are highly sensitive to heat, light, and oxygen [65,98]. Their volatility, hydrophobicity, irritancy, and instability limit their direct application in food packaging. To preserve their structure and activity, essential oils typically require encapsulation. Essential oils can be encapsulated in gelatin matrix. Gelatin not only provides a controlled and safe delivery environment, but also improves the bioavailability and stability of essential oils. Meanwhile, the hydrophobicity of the gelatin-based film is enhanced. This encapsulation imparts additional antibacterial, antioxidant, and functional properties to the film [99].

In recent years, gelatin films modified with plant essential oils have been increasingly utilized in the development of pH sensors due to their eco-friendly, biodegradable, non-toxic, antibacterial, and antioxidant properties [100]. Pickering emulsion composed of alizarin and oregano essential oil (OEO) was introduced into gelatin films, resulting in a novel multifunctional food packaging [101]. The incorporation of OEO enhanced the UV resistance and mechanical properties of the film. The controlled release effects of the Pickering emulsion significantly improved the film’s antioxidant and antibacterial capabilities. The film exhibited a distinct color change from yellow to purple as the pH increased within the range of pH 3–11 and demonstrated sensitivity to ammonia vapor within four minutes. This innovative gelatin-based film slowed the spoilage of beef and provided real-time freshness monitoring through color changes.

However, the direct addition of essential oils to the gelatin film-forming solution can lead to phase separation, which typically increases film unevenness and surface roughness, thereby reducing transparency. Consequently, during the preparation of the gelatin/essential oils composite film-forming solution, it is essential to emulsify the oil phase prior to casting the film. Compared to pure gelatin films, gelatin composite films containing natural essential oils exhibit a significant reduction in light transmittance due to the migration of oil droplets to the film’s surface during the drying process.

#### 3.1.4. Tannins

Tannins (TAs) are natural polyphenolic compounds widely distributed in plants [102]. Under oxidative conditions, TAs react with the amino groups of peptide side chains, leading to crosslinking within gelatin molecules. Due to the high content of proline in gelatin, TAs exhibit a strong affinity for gelatin. Tannin-crosslinked gelatin films demonstrate excellent mechanical strength and stability. TAs have long been utilized as natural crosslinking agents in the food industry [103,104,105,106,107]. TA was incorporated into gelatin-silver nanocomposite films, significantly improving the films’ water vapor barrier [64]. The presence of TAs also increased the mechanical strength and thermal stability of the films, contributing to the maintenance or extension of the shelf life of packaged foods. Grape seed TA (Seed T) and grape skin TA (Skin T) can enhance the properties of gelatin films for smart packaging [108]. The addition of these two types of TA resulted in gelatin films with low wettability and high UV shielding capability. Furthermore, the introduction of TAs significantly enhanced the antioxidant properties of the gelatin films. Notably, gelatin films containing grape skin TA (Skin T) exhibited pH-sensitivity.

#### 3.1.5. Synthetic Degradable Materials

Many bioplastics incorporate synthetic degradable polymeric materials that enhance product functionality and broaden applications. Similarly, the incorporation of renewable materials into gelatin can address the strong hydrophilicity and poor mechanical properties of gelatin films, leading to the development of biodegradable smart packaging.

Polyvinyl alcohol (PVA) is a non-toxic polymer known for its excellent chemical stability and biocompatibility. The abundance of hydroxyl groups in its structure facilitates the formation of hydrogen bonds during the film-forming process, resulting in good film-forming [109]. Consequently, PVA is widely utilized in fields such as biomedicine [110,111,112,113], food packaging [114,115,116,117,118], and the coatings industry [119,120,121,122]. As a bioindicator, amaranth leaf extract (ALE) was introduced a combination of PVA and gelatin to prepare an active film [123]. The reduction in both water solubility and water vapor permeability of the film, while significantly enhancing its mechanical properties, thereby rendering it suitable as a smart packaging material for meat. Redox polymerization technology was employed to incorporate N,N-dimethylacrylamide (DMAAm) and pomegranate extract into gelatin, resulting in a biocompatible gelatin-based smart hydrogel [124]. The findings demonstrate that DMAAm enhanced both the mechanical properties and water resistance of the hydrogel, which also exhibited excellent thermal stability, antibacterial activity, and pH sensitivity, making it a promising candidate for smart food packaging.

Blending gelatin with synthetic degradable materials can significantly improve the mechanical properties, water resistance, and stability of gelatin-based films. Unlike conventional packaging, which primarily provides sealing and preservation functions, these multifunctional films offer comprehensive protection against microorganisms, oxygen, and ultraviolet light, thus extending the shelf life of food. However, the potential toxicity of these gelatin-based composite materials remains a topic of ongoing debate. The challenge ahead lies in the selection of nontoxic synthetic degradable materials to achieve effective multifunctional modifications of gelatin-based products.

### 3.2. Intelligent Indicators

**Figure 3:**
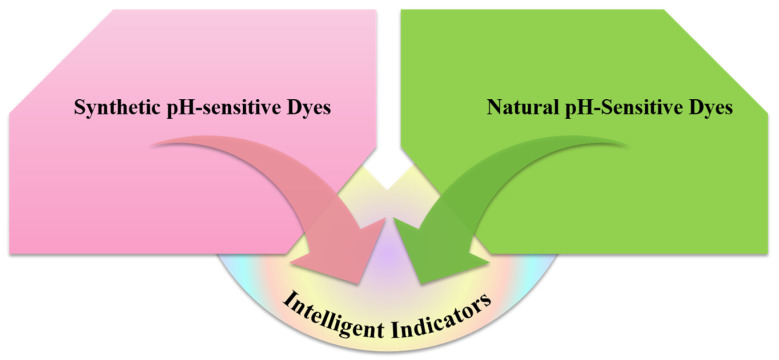
Types of pH-sensitive dyes.

The primary function of the intelligent indicators is to detect and communicate critical information regarding product freshness, safety, and storage conditions. It responds to environmental changes within the packaging and provides real-time, non-invasive, visual information on food quality through colorimetric responses during storage and transportation [125]. The pH-sensitive indicators typically incorporates one or more pH-sensitive dyes that detect specific compounds and environmental changes within the packaging. These dyes convey information on food freshness or microbial quality to consumers by means of visible color changes [126,127]. As illustrated in Figure 3, pH-sensitive dyes are categorized into two types, synthetic pH-sensitive dyes and natural pH-sensitive dyes. The careful selection of an appropriate pH-sensitive dye is crucial for the effective development of gelatin-based pH sensors.

#### 3.2.1. Synthetic Dyes

Synthetic pH-sensitive dyes were the first to be utilized for color indication in pH sensors. Acid–base indicators, such as bromocresol purple [128], bromophenol blue [129], bromocresol green [130], chlorophenol red [131,132], cresol red [133,134], and bromocresol green [135], have been used in food packaging to provide real-time information on food quality [52,136]. Three synthetic indicators (methyl orange (MO), neutral red (NR), and bromocresol green (BCG)) were incorporated into a gelatin matrix to develop color-changing gelatin films that communicate food quality through visual color variation [40]. The resulting gelatin films demonstrated pH responsiveness, displaying different colors when exposed to media of varying pH levels (liquid, semi-solid, or gas). Moreover, the inclusion of MO and NR enhanced the mechanical properties of the gelatin films while simultaneously reducing their water solubility.

However, concerns regarding food safety have raised significant issues about the use of synthetic pH-sensitive dyes. These dyes are potentially toxic and pose both health and environmental risks, which has led to controversy over their application in pH sensors. Consequently, their practical application has been limited, and they are increasingly being replaced by natural pH-sensitive dyes [137].

#### 3.2.2. Natural Dyes

Natural pH-sensitive dyes are increasingly favored over synthetic alternatives to meet growing market demands for safer and more eco-friendly packaging. These natural dyes are abundant, easily accessible, and widely distributed in various colorful fruits, vegetables, and other plants [138]. Dyes such as anthocyanins [139], carotenoids [140], betalains [141], curcumin [142], and chlorophyll [143] have been extensively studied for incorporation into pH sensors. The structures of these natural dyes typically contain multiple conjugated phenolic rings. When environmental pH changes occur, the phenolic hydroxyl groups undergo protonation or deprotonation, altering the molecular coupling patterns and resulting in visible color changes [144].

Natural dyes offer numerous advantages, including safety, a broad signal range, and strong pH responsiveness. Studies have demonstrated that natural dyes not only effectively indicate changes in food quality, but also possess antimicrobial and antioxidant properties, which can help extend the shelf life of foods [145,146,147]. By utilizing gelatin as a substrate and incorporating various natural dyes into the gelatin matrix, it is possible to develop multifunctional gelatin-based pH sensors that combine smart sensing capabilities with antimicrobial and antioxidant functionalities.

Compared to synthetic dyes, natural dyes present several advantages, including non-toxicity, renewability, and environmental compatibility. However, it is essential to recognize that crude plant extracts often contain other polyphenolic compounds, which may interfere with the colorimetric responses of natural dyes. As a result, crude plant extracts cannot be directly utilized in the preparation of gelatin-based pH sensors and must undergo purification prior to use [138]. Furthermore, the color range, sensitivity, permeability, and stability of natural dyes as food indicators within the gelatin matrix still require further optimization [137].

## 4. Summary and Outlook

Novel gelatin-based pH sensors are poised to shape the future of food packaging. In recent years, gelatin-based pH sensors have gained significant traction in the field of active smart packaging due to their unique advantages, including sensitivity, safety, rapid response, and non-invasiveness [15]. To be widely adopted, these materials must be cost-effective, sensitive, easy to prepare, and capable of real-time monitoring of food quality using visible color changes [148]. Table 2 provides a comprehensive summary of recent advancements in food freshness monitoring utilizing gelatin-based pH sensors, highlighting their performance characteristics and application potential. Future research should focus on the following key areas.

(1) Enhancing the reproducibility of gelatin-based pH sensors. When interfacing with high-moisture food surfaces, gelatin films demonstrate significant moisture absorption properties, leading to substantial water uptake. This pronounced hygroscopic behavior induces the rapid swelling of the film matrix, resulting in considerable alterations to both its structural integrity and functional properties. Despite the widespread application of gelatin in pH sensors development, the major challenge with gelatin films is their elevated water solubility and moisture sensitivity together with their weak mechanical strength and poor stability [150]. The weak mechanical properties and poor stability of gelatin film limit its applications in food packaging. Additionally, gelatin sourced from various origins exhibits differing molecular structures and functional characteristics, which may lead to inconsistent reproducibility in gelatin-based pH sensors. At present, key issues include achieving rapid and safe functional modifications while simultaneously enhancing the stability of gelatin film, particularly its thermal stability and mechanical stability. Optimizing these characteristics is essential for producing gelatin-based pH sensors with extended operational lifetimes, improved sensitivity, and reliable field performance. Therefore, safe and efficient functional modifications are necessary to meet specific application requirements. Detailed studies on design parameters and component miscibility during the modification process are critical. Exploring efficient, low-cost, and environmentally friendly modification methods will be essential to improve the performance and applicability of gelatin-based pH sensors. Addressing these challenges will be crucial for achieving the long life, high sensitivity, and in-field application of gelatin-based pH sensors.

(2) Improving the stability of natural dyes. Maintaining the stability of natural dyes embedded in multifunctional gelatin-based films is challenging due to interactions between the gelatin matrix and active compounds. These dyes are highly sensitive to environmental factors such as light, temperature, and pH, which can compromise the visual appearance and accuracy of gelatin-based pH sensors [151]. Future research should prioritize enhancing the stability of natural dyes under various storage and transportation conditions. Additionally, structural design innovations in gelatin-based smart materials could the controllable release kinetics of active substances, ensuring sustained functionality throughout the entire shelf life of food.

(3) Optimizing the pH sensitivity and compatibility of the pH sensors. Gelatin-based pH sensors can provide qualitative or semi-quantitative information about food quality changes while preserving the integrity of the packaging, allowing consumers to visually assess freshness. However, gelatin’s composition includes various α-amino acids with ionizable side groups that may act as a buffering system, potentially affecting the films’ responsiveness to pH changes. Furthermore, these side groups can exhibit high reactivity towards crosslinking or grafting, which may impact the sensitivity of the gelatin-based pH sensors [40]. As a result, these systems are not universally applicable. Their compatibility with the specific food being monitored must be confirmed. Optimal functionality is achieved only when the gelatin-based pH sensors is tailored to the appropriate type of food. Additionally, while these systems monitor a single parameter (pH), they cannot provide comprehensive insights into overall food quality. Monitoring processes may also face errors or malfunctions, and variations in storage and transportation conditions could further compromise sensitivity and stability. Therefore, further in-depth studies on the structural stability of gelatin-based pH sensors under diverse environmental conditions are essential to ensure consumer health and safety.

(4) Achieving commercial applications of the Gelatin-based pH sensors. Gelatin-based pH sensors offer significant advantages for food packaging applications as cost-effective, power-free quality indicators that provide visual readouts interpretable by consumers without technical training. Despite these benefits, the development of gelatin-based pH sensors remains at an early stage with substantial untapped potential [152]. While their production requires minimal specialized equipment and reduced material inputs compared to conventional sensors, current formulations still face inadequate long-term stability and the susceptibility of colorimetric indicators to environmental degradation. These constraints render the conventional casting approach unsuitable for commercial-scale manufacturing. Although considerable research efforts have been directed toward developing novel gelatin-based pH sensors with enhanced environmental stability and reduced sensitivity to external factors, the majority of current research remains confined to laboratory-scale investigations, with limited translation to commercial applications. These challenges represent a critical research gap that requires urgent attention in future. Bridging this gap will require concerted efforts to optimize manufacturing processes while maintaining the functional integrity and cost-effectiveness of gelatin-based sensors.

By addressing these challenges, gelatin-based pH sensors can achieve broader applicability and reliability, paving the way for their widespread adoption in the food industry.

## Figures and Tables

**Table 1:** Market share of gelatin from different sources.

Sources of Gelatin	Market Share (%)
Pig skin	40%
Pig Cartilage	6%
Bovine hides	29.4%
Beef bones	23.1%
Other sources (poultry, fish, vertebrates, and so on)	1.5%

**Table 2:** Application of gelatin-based pH sensors for food freshness monitoring.

Active Carriers	pH-Sensitive Dye	Food Samples	Storage		Color Change	pH		Sensitivity	Ref.
			Time	Temp. (°C)		Initial (Fresh)	Final (Spoiled)	ΔE Value	
Gelatin, Chitosan, ZnO-nanoparticles	Mulberry extract (ME)	Pork	8 d	4 °C	red to blue/green	5.7	6.5	30.55	[94]
Gelatin, Oxidized chitin nanocrystals(O-ChNCs)	Black rice bran anthocyanins	Shrimp, Hairtail	1 d	25 °C	rose-carmine to yellow-green	7.5	8.2	7.27–10.47	[95]
Gelatin, Dialdehyde starch (DS)	Rosmarinic acid (RosA), Blueberry anthocyanins extract (BAE)	Fish	24 h	25 °C	red to brown to dark brown	7.0	8.2	10.23	[149]
Gelatin, Carrageenan	Shikonin extracted from the gromwell (Lithospermum erythrorhizon) root	Milk	-	25 °C	bright red to blue	6.6	4.5	55.7	[76]
Gelatin, Starch	Red cabbage extracts	Milk	-	25 °C	red to green/yellow	6.8	4.0	-	[83]
gelatin, Polyvinyl alcohol (pva)	Amaranthus leaf extract	Chicken	12 d	2–4 °C	pink to yellow	5.5	8.6	22.13	[123]
Gelatin, Bacterial cellulose nanofibers	Curcumin/Anthocyanin (Cur/ATH)	Pork	4 d	4 °C	yellow to red	5.7	6.7	55.83	[145]
Gelatin hydrolysate (GELH), Furcellaran (FUR),	Rosemary extract from dry leaves (DRE))	Carp (Cyprinus carpio)	14 d	4 °C	green to red	5.8	7.0	56.67	[77]
Gelatin, Lavender essential oil	Alizarin (ALI)	Shrimp	3 d	25 °C	yellow to brown-red	7.5	8.2	55	[100]

## Data Availability

Not applicable.
